# Clinical Management of Malignant Insulinoma: a single Institution’s experience over three decades

**DOI:** 10.1186/s12902-018-0321-8

**Published:** 2018-12-06

**Authors:** Jie Yu, Fan Ping, Huabing Zhang, Wei Li, Tao Yuan, Yong Fu, Kai Feng, Weibo Xia, Lingling Xu, Yuxiu Li

**Affiliations:** Department of Endocrinology, Chinese Academy of Medical Sciences and Peking Union Medical College, Peking Union Medical College Hospital, Beijing, China

**Keywords:** Malignant insulinoma, Metastasis, Hyperinsulinism, Hypoglycemia, Diagnosis, Therapeutics

## Abstract

**Background:**

Malignant insulinoma is extremely rare and accounts for only 10% of total insulinoma cases. The goal of this study is to retrospectively analyze clinical data from 15 patients with malignant insulinoma treated at Peking Union Medical College Hospital (PUMCH) from 1984 to April 2017.

**Methods:**

“Malignant insulinoma” was used as the keywords in the PUMCH medical record retrieval system to search and obtain patients’ clinical information. We identified subjects diagnosed with malignant insulinoma based on clinical or surgical pathological signs and subsequently analyzed their clinical data.

**Results:**

Eight males and seven females with a median age at diagnosis of 40 years (38–54 years) were included. Eight patients (53%) had developed metastases at diagnosis, while the others (46.67%) developed metastases during the follow-up visits. The major sites of metastasis were the liver (86.7%), local tissues and blood vessels (33%) and abdominal lymph nodes (13%). All patients displayed neuroglycopenic (100%) and/or autonomic (60%) symptoms, mostly during fasting periods (73.3%), with an average blood glucose level of 1.66 ± 0.51 mmol/L. A total of 93% of the patients had one primary pancreatic lesion, 53% had a lesion in the head of the pancreas, and 47% had a lesion in the tail of the pancreas, with diameters ranging between 0.9 and 6.0 cm. Most liver metastases were multiple lesions. Selective celiac arteriography yielded 100% sensitivity for both primary pancreatic lesions and liver metastases. Most patients received synthetical treatments, including surgery, chemoembolization, and octreotide.

**Conclusions:**

Malignant insulinomas have a similar diagnostic process to that of benign insulinomas but require far more comprehensive therapies to alleviate hypoglycemic symptoms and extend patients’ survival.

## Background

Insulinoma is a type of functional pancreatic neuroendocrine tumor (pNET) that originates from islet beta cells, which excessively secrete insulin and cause hypoglycemia. Insulinoma is the most common functional pNET, with a prevalence of approximately one to four cases per million people [1]. Four features of insulinoma are associated with four “90%” parameters: 90% are benign, 90% are solitary, 90% occur in the pancreas, and 90% are less than 2 cm in diameter [1]. Malignant insulinoma is extremely rare and accounts for only about 10% of all insulinoma cases [2]. Malignant insulinoma refers to cases exhibiting local invasion or distal metastasis, which is the only biological property that differentiates malignant cases from benign cases. Clinical manifestations, biochemical traits, and pathology cannot be used as direct evidence for identification. Benign insulinomas are typically treated surgically, whereas malignant insulinomas often require comprehensive approaches to maximize patient survival. In this study, we analyzed the clinical data of 15 cases of malignant insulinoma treated at the Peking Union Medical College Hospital (PUMCH) from 1984 to April 2017.

## Methods

### Study subjects

“Malignant insulinoma” was used as the keywords to search the PUMCH medical record retrieval system for patients with a discharge diagnosis of malignant insulinoma and their related clinical data from 1984 to April 2017. The search yielded 25 patients. Among these patients, the medical records of three patients were unavailable, two patients were later diagnosed with benign insulinoma, two patients were diagnosed with recurrent insulinoma, two patients were diagnosed with multiple insulinomas, and one patient had no clear diagnosis; therefore, 15 patients diagnosed with malignant insulinoma based on clinical and/or pathological evidence were included in this study. The clinical diagnostic criteria for malignant insulinoma included the following: i) hypoglycemic symptoms and the Whipple triad; ii) endogenous hyperinsulinemic hypoglycemia symptoms, with a blood glucose level < 3 mmol/L, insulin level > 3 μIU/mL, and C-peptide level > 0.6 ng/mL; and iii) pancreatic tumors with local infiltration or distant metastases based on evidence from one or several imaging methods. In addition, the pathological diagnostic criteria included a clinical diagnosis with surgical or puncture specimens indicative of a pNET [3].

### Study methods

#### Retrieval of clinical information

We retrospectively analyzed the detailed clinical information of the 15 patients, including 1) general information, such as gender, age at disease onset, age at diagnosis, family history, and the presence of multiple endocrine neoplasia; 2) clinical signs, such as the time of hypoglycemia onset and neuroglycopenic and autonomic symptoms at hypoglycemia onset; and 3) qualitative and localization diagnostic information; the qualitative diagnosis included the blood glucose, insulin, and C-peptide levels at hypoglycemia onset, and localization methods included abdominal ultrasound, abdominal enhanced computed tomography (CT), pancreatic volume perfusion CT, octreotide imaging, and selective celiac arteriography; 4) treatments, including surgery, interventional approaches, chemotherapy, and somatostatin analogues; 5) tumor characteristics, including the number, size, distribution, and location and metastasis information; and 6) pathological information. Most patients did not have detailed histological information, and we could not perform tumor staging or analyze the Ki-67 index. Moreover, we divided the 15 patients into two groups according to the timing of metastasis diagnosis: metastasis upon diagnosis or metastasis during the follow-up. Then, some clinical characteristics were compared between the groups. All follow-up visits were performed by phone calls in which the patients or their family members stated the disease conditions.

#### Biochemical assays

##### Glucose detection

The glucose oxidase assay was performed to determine the serum glucose level. The serum insulin and C-peptide levels were examined using radioimmunoassays (DPC, America) prior to 1991 and chemiluminescence assays (ADVIA Centaur XP, Siemens) after 1991. All tests were performed in the Clinical Laboratory of the PUMCH.

### Data analysis

Continuous data are expressed as the mean ± standard deviation when they were normally distributed or as the median (interquartile range) when they were not normally distributed. A t test was performed to compare the means of the continuous data with a normal distribution, whereas the Mann-Whitney U test was used for continuous data with a non-normal distribution. Statistical analyses were performed using IBM SPSS Statistics Version 22.0 (Chicago, IL, USA). A two-tailed *P* < 0.05 was considered significant.

## Results

### Basic characteristics

The 15 patients included eight males and seven females with a median age at diagnosis of 40 (38–54) years. Eight patients (53%), including four males and four females, had metastases upon diagnosis, whereas the seven remaining patients, including four males and three females, developed hepatic metastases 2 to 25 years after the insulinoma diagnosis. The most common site of metastasis was the liver (86.7%), followed by local tissues and blood vessels (33%) and abdominal lymph nodes (13%). Two patients (13%, Nos. 9 and 15) experienced hepatic metastases and pancreatic local recurrence during the follow-up visits (Table 1).

**Table 1:** Clinical characteristics of the 15 patients with malignant insulinoma

No^c^.	Gender	Age at diagnosis	Hypoglycemia time	GLU (mmol/L)	INS (μIU/mL)	C-P (ng/mL)	Number and diameter (cm) of primary lesions	Location of primary lesions^a^	Local infiltration	Sites and numbers of metastases	Treatment after metastasis occurrence	Prognosis	Follow-up duration (years)^b^
1	F	54	Fasting, before meals	1.4	32.05	5.6	1, 5 × 5	Head	Yes	Multiple liver metastases	TACE for 1 course	N. A.	Loss to follow-up
2	F	63	Fasting	1.3	81.41	3.9	1, 1.8 × 1.4	Neck	No	Multiple pulmonary metastases, bone metastases?	Automatic discharge	No symptoms of hypoglycemia	Survival (7 years)
3	M	46	Fasting	1.6	62.52	5.2	1, 5.8 × 4.3	Tail	Yes	Multiple liver and lymph nodes metastases	Somatostatin analogues for 9 courses	Alleviation of hypoglycemia	Loss to follow-up
4	M	51	Before meals	1.0	N.A^d^.	N.A.	3, 3 × 4	Tail	Yes	Multiple liver metastases	Distal pancreatectomy	No remission	Loss to follow-up
5	M	26	Fasting	2.2	121.8	N.A.	1, 1.5	Head	No	Multiple intracranial metastases	Pancreatic lesion enucleation	Blood glucose increased, still in a coma	Loss to follow-up
6	F	30	Before meals	1.0	300	7.9	1, 0.9	Body	No	Multiple liver and lymph nodes metastases	Enucleation with partial hepatectomy, Sandostatin LAR for 2 courses	No hypoglycemia	Survival (0.8 years)
7	M	55	Fasting	1.9	148	N.A.	1, 6	Tail	Yes	Multiple liver metastases	Exploratory laparotomy, TACE for 1 course	N.A.	Loss to follow-up
8	F	39	Fasting, after meals and exercise	1.9	15.2	2.9	1, N.A.	Head	No	Multiple liver metastases	pancreatic surgery (unspecified)	No hypoglycemia	Survival (14 years)
9	M	72	Fasting, before meals	2.3	29.48	2.7	1, 1.5	Head	No	Solitary liver metastases	Surgical resection of liver metastases	No remission	Survival (4 years)
10	F	39	Before meals	1.5	42.2	2.5	1, 1.2	Head	No	Multiple liver metastases	Exploratory laparotomy, absolute ethanol injection ablation for 3 courses, TACE for 10 courses	Temporary remission	Survival (13 years)
11	M	40	Fasting	2.9	5.2	0.6	1, 0.8 × 1	Head	No	Multiple liver metastases	Sandostatin LAR for 2 courses	No remission	Loss to follow-up
12	F	50	Fasting, before meals, after exercise	1.9	300	21.61	1, N.A.	N.A.	No	Multiple liver metastases	Liver interventional therapy for 4 courses, liver surgery (unspecified)	No hypoglycemia	Survival (6 years)
13	M	38	Fasting, after exercise	2.1	N.A.	N.A.	1, 1	Tail	No	Solitary liver metastases	Surgical resection of liver metastases	No hypoglycemia	Loss to follow-up
14	M	36	Fasting	0.9	17	3.7	1, N.A.	Neck	No	Multiple liver metastases	Exploratory laparotomy, TACE for 1 courses	N.A.	Loss to follow-up
15	F	38	Fasting, before meals	1.6	71.8	7.3	1, 6 × 4	Tail	No	Multiple liver metastases	TACE for 4 courses	3 years of remission	Loss to follow-up

^a^Location of primary lesions: final determination was based on preoperative mapping and intraoperative positioning

^b^Survival duration: the period between metastasis occurrence and follow-up visits

^c^Case Nos. 1–8 had developed metastasis upon diagnosis, whereas case Nos. 9–15 developed metastasis during the follow-up visits

^d^N.A., data not available

### Clinical manifestations

All 15 subjects showed symptoms of hypoglycemia manifesting as neuroglycopenic symptoms (100%) and/or autonomic symptoms (60%). The most common neuroglycopenic symptoms included confusion (80%), coma (46.7%), behavioral changes (46.7%), and seizure (40%). The most common autonomic symptoms included sweating (53.3%), weakness (40%), palpitations (33.3%), and hunger (20%). Symptoms other than hypoglycemia were rare, and only one patient (No. 1) developed repeated back pain at disease onset, while one patient (No. 12) presented with discomfort under the xiphoid process and in the right abdomen at the time of liver metastasis diagnosis at a follow-up visit.

Hypoglycemia most often occurred during periods of fasting (73.3%), followed by before lunch or before dinner (46.7%), whereas hypoglycemia was rare after meals (6.7%). The average blood glucose level upon symptom onset was 1.66 ± 0.51 mmol/L.

Patient No. 11 additionally presented with asymptomatic primary hyperparathyroidism and was clinically diagnosed with multiple endocrine neoplasia type 1 (MEN1). Patient No. 8 additionally presented with left adrenal nonfunctional adenoma and was clinically suspected of having MEN1. Neither of the two cases had a family history of MEN1 or received the MEN1 gene test.

### Biochemical analysis and lesion localization

All patients denied previous use of sulfonylureas or insulin. Eleven patients had documented blood glucose, insulin, and C-peptide levels (Table 1), which were all consistent with the criteria for endogenous hyperinsulinemic hypoglycemia. A comparison between the patients with metastases upon diagnosis and their counterparts who developed metastases during the follow-up revealed that the former group appeared to have lower glucose levels but higher insulin and C-peptide levels than the latter group, although the differences were not significant (Table 2). Ten patients had histological confirmation of a pancreatic primary lesion or liver metastatic lesion through surgical or puncture biopsy pathology.

**Table 2:** Blood glucose, insulin, and C-peptide concentrations with hypoglycemia

	Metastasis identified upon diagnosis	Metastasis occurred during follow-up	*P value*
Glucose (median, quartile, mmol/L)	1.5 (1.1–1.9)	1.9 (1.5–2.3)	0.23
Insulin (median, quartile, μIU/mL)	81.41 (32.05–148.00)	35.84 (14.05–128.85)	0.28
C-peptide (median, quartile, ng/mL)	5.20 (3.40–6.75)	3.20 (2.03–10.88)	0.36

All patients except for one had a solitary pancreatic primary lesion. The maximal diameters of the primary lesions ranged between 0.9 and 6.0 cm, 53% of the lesions were located in the head and neck, and 47% of the lesions were located in the body and tail. The most common metastatic site was the liver (86.7%), and almost all cases of liver metastasis (84.6%) involved multiple hepatic metastases. Among the eight patients with metastasis at diagnosis, tumors were evenly distributed in the head, body, and tail of the pancreas; however, among the seven patients who developed metastasis during the follow-up visits, 50% of the tumors were in the head of the pancreas (Table 1).

Spearman analysis was performed to test the associations between the tumor size and insulin, C-peptide, and glucose levels. We have found a positive correlation between tumor size with both insulin and C-peptide levels(*r = 0.183, P = 0.613; and r = 0.268, P = 0.493, respectively*), but a negative correlation between tumor size and glucose levels (*r = − 0.182, P = 0.572*), although none of the differences reached significance due to the small sample size.

### Sensitivity of localization tests

Of the 10 patients with a histologically confirmed pancreatic primary lesion or liver metastatic lesion, 6 patients received selective celiac arteriography 7 times, and the sensitivity for both primary pancreatic lesions and liver metastases was 100%. Next, we used celiac arterial angiography or pathological confirmation as the gold standard to analyze the sensitivity of other preoperative noninvasive localization examinations, as shown in Table 3.

**Table 3:** Sensitivity of preoperative noninvasive localization tests

	Primary lesions		Liver metastases	
	Cases examined	Sensitivity	Cases examined	Sensitivity
Abdominal ultrasound	10	50%	6	85.7%
Abdominal enhanced CT	7	50%	6	83.3%
Pancreatic volume perfusion CT	2	50%	2	100%
Octreotide imaging	1	0	2	0

### Treatment and efficacy

Of the eight patients with metastases at diagnosis, four patients underwent surgery, three of whom displayed total or partial remission of hypoglycemia (75%) (Table 1). Among the other four patients who did not undergo surgery, one rejected any treatment, two underwent transcatheter arterial (chemo) embolization (TAE/ TACE) but were later lost to follow-up, and one patient (Patient No. 3) received somatostatin analogues (five treatments of Sandostatin LAR and four treatments of Somatuline), which alleviated hypoglycemic symptoms.

Of the seven patients who showed no metastases at diagnosis, five patients underwent enucleation, one patient (Patient No. 13) underwent distal pancreatectomy and splenectomy, and one patient (Patient No. 11) underwent complete pancreatectomy; all patients achieved complete remission of hypoglycemia. During the follow-up, two of these patients displayed a single liver metastasis, and resection of the liver lesion relieved hypoglycemia in only one of these patients (50%). Of the other 5 patients who displayed multiple liver metastases, one underwent surgery combined with TAE/ TACE, which resulted in complete remission of hypoglycemia, one patient received somatostatin analogues, and three patients received only TAE/ TACE, resulting in only temporary remission or no remission (Table 1).

Because the availability of diazoxide was limited in hospitals and pharmacies in mainland China, only one patient (Patient No. 10) received diazoxide in a short period of time, but the drug was discontinued due to poor performance.

### Follow-up visits

Of the 15 patients, six completed the follow-up visits, with a duration ranging from nine months to 29 years. All six of these subjects survived. The period from metastasis occurrence to follow-up ranged from nine months to 14 years, with a median of 6.5 years. Among these patients, five (83.3%) were females with a median diagnostic age of 42.5 (30–47) years. Three patients had metastases at diagnosis, whereas the other three showed metastases during the follow-up visits. All patients had a single primary pancreatic lesion with a maximal diameter of 0.9–1.8 cm. Four patients had multiple liver metastases, and one patient had multiple pulmonary metastases and suspected bone metastases, while the other one patient successively developed a single hepatic metastasis and pancreatic recurrence in situ. Five patients underwent cytoreductive surgery and/or combined with TAE/ TACE and/or somatostatin treatment. Currently, four patients have no hypoglycemic symptoms, and two patients need extra meals every day (Table 1).

## Discussion

Insulinoma is the most common functional pancreatic neuroendocrine tumor (pNET), and malignant insulinoma is very rare, with a prevalence of only one case per million people [4], accounting for 7–10% of all cases of insulinoma [1, 5]. In this study, we retrospectively analyzed the clinical data of 15 cases of malignant insulinoma treated at the PUMCH from 1984 to April 2017. The numbers of males and females were similar, and the median age at diagnosis was 40 (38–54) years. More than 50% of the patients exhibited metastases at diagnosis, with the liver as the most common metastatic site. The main clinical manifestations were hypoglycemia-related symptoms. Unlike patients with benign insulinoma, those with the malignant type exhibit pronounced neuroglycopenic symptoms. Selective celiac arteriography yielded 100% positive rates for both pancreatic primary lesions and liver metastases. Almost all patients had solitary lesions of different sizes in the pancreas, which were evenly distributed in the pancreas. The therapeutic approaches included surgery, TAE/ TACE, and somatostatin analogue administration.

Malignant insulinoma has a reported onset age of 50–60 years, which is older than the ages of the patients in our report, and no gender preference [2]. Affected patients mostly exhibit neuroglycopenic symptoms, resulting from higher insulin and proinsulin secretion, which is consistent with our study. Although, the severity of hypoglycemia symptoms is reportedly not proportional to the tumor burden, we found a positive correlation between tumor size and insulin and C-peptide level [2, 6].

Liver metastasis upon disease onset is the most common manifestation of malignant insulinoma. Rarely, malignancy is diagnosed at the time of recurrence, which occurs for only 2% of insulinomas overall [2]. The most common metastatic sites of malignant insulinoma are the abdomen, including the retroperitoneal lymph nodes and liver, while bone, lung or other metastasis sites are rare [1, 2, 7]. In our study, more than 80% of the patients displayed liver metastasis, more than 30% of the patients showed invasion into local blood vessels or neighboring tissues, only one patient had brain metastasis, and one patient showed lung metastasis and suspected bone metastasis, which are consistent with previous studies.

Among noninvasive localization methods for insulinoma, abdominal ultrasound had a relatively low sensitivity of 9–64% [8], CT and magnetic resonance imaging (MRI) yielded a sensitivity of 56–70% and 63–86% separately [9, 10]. As for invasive approaches, sensitivity of endoscopic ultrasonography (EUS) was 86.6–92.3% [9], and arteriography had a sensitivity of approximately 70% [11]. In our previous study, we separately analyzed the sensitivity for primary pancreatic lesions and liver metastases, and found that arteriography had the best performance and all of abdominal ultrasound, enhanced CT and volume perfusion CT (VPCT) had considerable higher sensitivities for detecting liver metastases versus pancreatic lesions. As for somatostatin receptor imaging, it was reported to generate a positive rate of 30–50% [2, 12], and Jin et al. reported that it had an 85.7% sensitivity for malignant insulinoma, which was higher than for benign insulinoma (37.9%) [13]. In our study, because of only few cases detected, the results were all negative. Alternatively, if we used a clinical diagnosis of malignant insulinoma as the criterion, it produced a sensitivity of 80% (4/5) for pancreatic primary lesions and 50% (3/6) for liver metastases, which is consistent with previous results [13].

A comprehensive approach is needed to treat malignant insulinoma, which includes medication, surgery, and interventional therapy. For patients with advanced or metastatic lesions, cytoreductive surgery is conducive to control hormone secretion [2], although whether it can extend survival remains controversial. TAE/TACE is often used to manage liver metastases and has an effectiveness rate greater than 50% [3]. Therapy using somatostatin analogues has an objective remission rate (ORR) of less than 10% and a disease control rate (DCR) between 35 and 40% [3]. In our study, 7 patients underwent cytoreductive surgery when they developed metastases, 71.4% of whom displayed increased blood glucose levels and showed complete or partial remission of hypoglycemia. Therefore, surgery is an effective approach to control symptoms for patients with malignant insulinoma, although the curative outcome may differ from one patient to another. TAE/TACE and somatostatin treatment approaches generated remission rates lower than those reported in a previous study [14], and the discrepancy may be due to the small study population and high loss to follow-up rate.

Lepage et al. studied 81 patients with malignant insulinomas and found that they had a five-year survival rate of 55.6% [15]. A report from the Mayo Clinic following 13 cases of malignant insulinoma found that these patients had a 10-year survival rate of 29% [16]. No consensus has been reached regarding this discrepancy, although Ki67 was proposed to affect total survival [4]. Hirshberg et al. compared survivors of a long course of disease and patients with a poor prognosis and discovered that the two groups were almost indistinguishable in terms of their pathomorphology, insulin levels, and proinsulin levels and that neither liver metastasis nor lymph node metastasis was a factor that contributed to a poor prognosis [7]. Our study had relatively limited follow-up data and did not calculate time-dependent survival. However, one patient survived for 14 years from the discovery of metastasis, indicating that malignant insulinoma did not necessarily mean a poor prognosis and that patients may still have a long survival period [7].

### Strengths and limitations

This study has some strengths and limitations. The strengths include the long observation period analyzed and the comprehensive patient analysis. The main study limitations are those associated with the retrospective nature of the study, the small patient number, and the long enrollment period spanning over decades (1984–2017), which implies a lack of uniformity in the diagnostic criteria, imaging resources, and treatment options, as well as an absence of detailed histological information and a small number of patients who were followed up. Therefore, we could not perform tumor staging and grading, and analyze the survival rate.

## Conclusion

Malignant insulinoma is extremely rare. More than 50% of the patients had already developed metastases upon diagnosis, with the liver as the most common site. The main clinical manifestations are hypoglycemia-related symptoms. Unlike benign insulinoma, the malignant type causes prominent neuroglycopenic symptoms. Regarding the localization examination, selective celiac arteriography generated positive rates of 100% for both pancreatic primary lesions and liver metastases. Some malignant insulinoma tumors were initially diagnosed as solitary benigh pancreatic tumors, indicating that the two types of tumor were difficult to distinguish clinically. However, patients with malignant insulinoma may develop metastasis during follow-up visits. Therefore, long-term follow-up visits are critical for patients with insulinoma.

## Abbreviations

ASVS: arterial calcium stimulation with hepatic venous sampling
CT: computed tomography
DCR: disease control rate
EUS: endoscopic ultrasonography
MEN1: type 1 multiple endocrine neoplasia
MRI: magnetic resonance imaging
ORR: objective remission rate
pNET: pancreatic neuroendocrine tumor
PUMCH: Peking Union Medical College Hospital
TAE/TACE: transcatheter arterial (chemo) embolization
VPCT: volume perfusion CT

## References

[1] Okabayashi T, Shima Y, Sumiyoshi T, Kozuki A, Ito S, Ogawa Y, Kobayashi M, Hanazaki K (2013). Diagnosis and management of insulinoma. World J Gastroenterol.

[2] Baudin E, Caron P, Lombard-Bohas C, Tabarin A, Mitry E, Reznick Y, Taieb D, Pattou F, Goudet P, Vezzosi D (2013). Malignant insulinoma: recommendations for characterisation and treatment. Ann Endocrinol (Paris).

[3] Xu J, Liang H, Qin S (2016). L W: expert consensus on gastrointestinal pancreatic neuroendocrine tumors in China (2016 version). Chin Clin Oncol.

[4] Giuroiu I, Reidy-Lagunes D (2015). Metastatic insulinoma: current molecular and cytotoxic therapeutic approaches for metastatic well-differentiated panNETs. J Natl Compr Cancer Netw.

[5] Iglesias P, Lafuente C, Martin AM, Lopez GA, Castro JC, Diez JJ (2015). Insulinoma: a multicenter, retrospective analysis of three decades of experience (1983-2014). Endocrinol Nutr.

[6] Wang L, Zhao Y, Chen G, Liao Q. The diagnosis and treatment of malignant insulinoma. J Hepatobiliary Surg. 2004:88–90.

[7] Hirshberg B, Cochran C, Skarulis MC, Libutti SK, Alexander HR, Wood BJ, Chang R, Kleiner DE, Gorden P (2005). Malignant insulinoma: spectrum of unusual clinical features. Cancer-Am Cancer Soc.

[8] ON T, PL C (2006). KC C: the management of insulinoma. Br J Surg.

[9] Goh BKP, Ooi LLPJ, Cheow P, Tan Y, Ong H, Chung YA, Chow PKH, Wong W, Soo K (2009). Accurate preoperative localization of Insulinomas avoids the need for blind resection and reoperation: analysis of a single institution experience with 17 surgically treated tumors over 19 years. J Gastrointest Surg.

[10] Zhu L, Xue H, Sun Z, Li P, Qian T, Xing X, Li N, Zhao Y, Wu W, Jin Z (2017). Prospective comparison of biphasic contrast-enhanced CT, volume perfusion CT, and 3 tesla MRI with diffusion-weighted imaging for insulinoma detection. J Magn Reson Imaging.

[11] Jackson JE (2005). Angiography and arterial stimulation venous sampling in the localization of pancreatic neuroendocrine tumours. Best Pract Res Cl En.

[12] Zhang T, Zhao Y, Cong L, Liao Q, Dai M, Guo J (2009). Noninvasive examinations for localization of insulinoma. Chinese Journal of Surgery.

[13] Jing H, Li F, Du Y, Long M, Xing X, Zhao Y, Niu N, Ma Y, Liu Y, Ba J, et al. Clinical evaluation of detecting insulinoma with 99mTc-HYNIC-TOC imaging. Chinese Journal of Medicine. 2012:35–7.

[14] Li X, Jin Z, Yang N, Liu W, Pan J. Transcatheter arterial chemoperfusion or chemoembolizaiton for treatment of liver metastasis from malignant insulinoma. Journal of Interventional Radiology. 2008:803–6.

[15] Lepage C, Ciccolallo L, De Angelis R, Bouvier AM, Faivre J, Gatta G (2010). European disparities in malignant digestive endocrine tumours survival. Int J Cancer.

[16] MM MM, O'Brien PC, Ballard DJ, Service FJ (1991). Functioning insulinoma--incidence, recurrence, and long-term survival of patients: a 60-year study. Mayo Clin Proc.

